# Computational models of autonomic regulation in gastric motility: Progress, challenges, and future directions

**DOI:** 10.3389/fnins.2023.1146097

**Published:** 2023-03-15

**Authors:** Omkar N. Athavale, Recep Avci, Leo K. Cheng, Peng Du

**Affiliations:** Auckland Bioengineering Institute, University of Auckland, Auckland, New Zealand

**Keywords:** gastroenterology, brain-gut axis, multi-scale modeling, electromechanical modeling, enteric nerves, vagus nerve, electrophysiology

## Abstract

The stomach is extensively innervated by the vagus nerve and the enteric nervous system. The mechanisms through which this innervation affects gastric motility are being unraveled, motivating the first concerted steps towards the incorporation autonomic regulation into computational models of gastric motility. Computational modeling has been valuable in advancing clinical treatment of other organs, such as the heart. However, to date, computational models of gastric motility have made simplifying assumptions about the link between gastric electrophysiology and motility. Advances in experimental neuroscience mean that these assumptions can be reviewed, and detailed models of autonomic regulation can be incorporated into computational models. This review covers these advances, as well as a vision for the utility of computational models of gastric motility. Diseases of the nervous system, such as Parkinson’s disease, can originate from the brain-gut axis and result in pathological gastric motility. Computational models are a valuable tool for understanding the mechanisms of disease and how treatment may affect gastric motility. This review also covers recent advances in experimental neuroscience that are fundamental to the development of physiology-driven computational models. A vision for the future of computational modeling of gastric motility is proposed and modeling approaches employed for existing mathematical models of autonomic regulation of other gastrointestinal organs and other organ systems are discussed.

## 1. Background physiology

Gastrointestinal (GI) motility is driven by complex interactions between nerves and myogenic electrophysiological mechanisms. Investigations have uncovered the physiological mechanisms of interaction between enteric nerves and interstitial cells of Cajal (ICC) as gastrointestinal pacemaker cells (Furness, 2012, 2022). *In silico* investigations of gastric motility have been used to bridge the understanding of the interactions between the neural and myogenic components of gastric motility and their implications in pathological developments. In particular, using simulations based on sound biophysical principles and specific experimental data, it is possible to simulate normal and pathological gastric electrophysiology and estimate biomarkers which match these scenarios. However, in these models the role of regulatory mechanisms, like the autonomic nervous system, has been omitted. Simplifying assumptions about the link between gastric motility and electrophysiology have been made in these models, however, with a burgeoning corpus of physiological research it is now possible to revise these assumptions and explore the impacts of autonomic regulation within a mathematical modeling framework (Du et al., 2013b,^2018^; Cheng et al., 2021).

The core of the ICC network exists in the myenteric (ICC-MY) and submucosal plexus (ICC-SM), with further intramuscular ICCs (ICC-IM) situated within the circular (ICC-CM) and longitudinal muscle layers (ICC-LM). ICC-IM are aligned with the muscle direction in their respective layers. ICC also exist in other GI organs, but their distribution varies. In general, one of the key physiological functions of ICC is to generate the major component of the gastric slow wave which controls rhythmic contractions of GI smooth muscle cells (SMCs) (Komuro, 1999; Kito, 2011). As a result, smooth muscle in the circular and longitudinal muscle exhibit coordinated depolarizations in the presence of ICC-MY, however in the absence of ICC-MY the depolarization of SMCs in the two layers is not coordinated (Huizinga, 2018). Gap junctions between cells of the gastric wall allow the flow of ionic currents between SMC, ICC, and PDGFRα+ cells in the stomach wall, forming a tissue structure that has been termed the SMC, ICC, PDGFRα+ syncytium (SIP syncytium) (Yeoh et al., 2016; Sanders, 2019).

Bioelectrical depolarization of SMC causes an influx of calcium ions and initiates a cascade of actions that lead to the contraction of muscle filaments (Hirst and Edwards, 2004). It is generally understood that gastric slow waves with inputs from the enteric nervous system (ENS) set the basic rhythmic contractions of the stomach. In the intact organ, the origin of the normal entrained slow waves is the pacemaker region along the greater curvature of the stomach. The waves propagate in coherent wavefronts in the aboral direction towards the pylorus. At the cellular level, the ICC network is believed to be responsible for setting the basic rhythmicity of slow waves and ensures that contractions are organized in an annular fashion (Mah et al., 2021). While ICC are known to exhibit decreasing intrinsic frequencies towards the pylorus of the stomach (Kelly et al., 1969), entrainment of slow waves occurs when the depolarization of a group of ICC with higher intrinsic frequency “phase locks” adjoining ICC with lower intrinsic frequencies, which results in a single frequency in the stomach, as well as over extended segments in the intestine (van Helden et al., 2010; Parsons and Huizinga, 2018). Persistent deviations of activities from the natural pacemaker region have been shown as a biomarker of diseases in gastroparesis and chronic nausea and vomiting (O’Grady et al., 2012; Angeli et al., 2015).

The brain-gut axis is used to label the plethora of interactions between GI organs and the brain. These interactions are mediated through the autonomic nervous system, of which the ENS is a part, and through the endocrine system (Lyte and Cryan, 2014). The effects of the brain-gut axis on the transport function of the stomach are covered in this work, but other functions of the stomach, such as secretion, are also influenced by the brain-gut axis. The vagus nerve is the primary, bi-directional communication pathway for the nervous system component of the brain-gut axis. Efferent vagal neurons originate at the brain stem and target cells in peripheral organs, and afferent vagal neurons originate at peripheral organs and target neurons in the brain stem. Observations made by microscopic imaging have shown that efferent vagal neurons release neurotransmitters at ENS ganglia, specifically targeting only a portion of a single ganglion (Powley et al., 2019). The ENS integrates extrinsic vagal input and intrinsic input from ENS interneurons, sensory neurons, and neuronal circuits thereby regulating gastric motility by neurotransmission to effector cells in the stomach wall (Furness et al., 2020). On the other hand, afferent vagal neurons are sensory neurons, chiefly sensing the stretch and strain of the stomach wall (Powley et al., 2019). Sensory neurons also exist within the ENS (Furness, 2012). Sensory neurons form feedback pathways to control stomach function, in conjunction with efferent nerves. Sensory neuronal activity is also a significant aspect of gastric neuro-circuitry but is not covered in detail in this review.

Extrinsic innervation of the stomach is chiefly provided by the vagus nerve though some sympathetic neurons originating in the T6-T9 level (thoracic) of the spinal cord innervate densely innervate gastrointestinal sphincters adjacent to the stomach (Browning and Travagli, 2014). Detailed quantification of the number and size of vagus nerve bundles was conducted by Prechtl and Powley (1985, 1990), with the measured diameter of neurons being consistent with the relatively slow conduction velocities observed in other parasympathetic nerves. Using cell tracer labeling with DiL and *in vivo* stimulation, Berthoud et al. (1991) deduced the branches of the vagus nerve from which innervation of gastrointestinal organs arises. Thoracic organs, such as the heart and lungs, are also innervated by the vagus nerve but branches to these organs diverge from the main vagus nerve at the cervical level.

The central nervous system influence on the upper gastrointestinal tract, including the stomach and esophagus, is complemented by reflexive control mediated at the level of the brainstem, termed vago-vagal reflexes. Afferent vagus nerve signaling to the nucleus tractus solitarius is integrated by neurocircuitry in the brainstem, and reflexes are relayed via efferent vagus neurons which originate at the dorsal motor nucleus of the vagus nerve. The gastric accommodation reflex results in a relaxation in gastric smooth muscle to accommodate food intake upon the distension of the proximal stomach. Another reflex, the esophagogastric reflex, results in gastric distension in response to stretch of the distal esophagus (Travagli and Anselmi, 2016). The nature of esophagogastric reflex disruption was noted as being similar between vagotomised patients and those diagnosed with functional dyspepsia (Troncon et al., 1995). The authors of the study suggested that vagus nerve defects may be responsible for some effect of functional dyspepsia.

The interface of neural and slow wave electrophysiology, with its complex interactions between multiple cooperating mechanisms, presents a complex challenge to the advance of *in silico* simulations. A previous review has covered aspects related to neuromodulation modeling of the gut (Barth and Shen, 2018), but there is significant knowledge gap and challenges to modeling the mechanistic components of automatic regulation. Specifically, the detailed interactions between sub-categories of ICC and the ENS. ICC have the ability to generate their own intrinsic slow waves and maintain an entrained wave propagation over an extended area of the GI tract, yet the influence of neural innervation of ICC over the same spatiotemporal scales remains incompletely understood (Forrest et al., 2006; Furness, 2012; Sanders and Ward, 2019). Furthermore, the ENS also has a level of direct control over GI smooth muscles, and it is unclear exactly how innervation interacts with slow waves under different physiological and patho-physiological conditions.

## 2. Mathematical models

A number of mathematical models have proposed to model various aspects of GI electrophysiology and motility (Du et al., 2010; Mah et al., 2021). The purpose of this section is to review approaches for continuum modeling of the stomach and their application in coupled-autonomic models; other reviews have provided more in-depth coverage of the different gastric function models (Du et al., 2010; Mah et al., 2021).

### 2.1. Cell electrophysiology models

**FIGURE 1:**
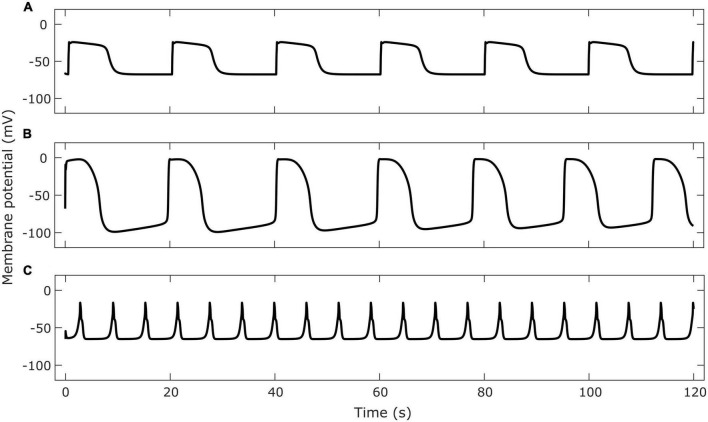
Examples of membrane potential simulated by cell models of gastrointestinal ICC. Cell model implementations were retrieved from the Physiome Model Repository. Panel **(A)** shows the membrane potential in a single gastric ICC-MY cell using the Corrias and Buist (2008) model. Panel **(B)** shows the membrane potential of a single small intestine ICC-MY using the Youm et al. (2006) model. Panel **(C)** shows the membrane potential of a single jejunal ICC using the Lees-Green et al. (2014) model.

The gastric slow wave is composed of multiple currents which are thought to originate in different cells (Sanders et al., 2006). Some models, (Edwards and Hirst, 2006; Faville et al., 2008; Youm et al., 2014) include detailed components that describe unitary potentials. A model of intracellular calcium by Means and Sneyd (2010) used stochastic terms to model IP_3_R activation. Models incorporating a variety of cellular pacemaking mechanisms have also been published. Youm et al. (2006) and Corrias and Buist (2008) modeled pacemaker potential initiation as a result of non-selective cation channel (NSCC) currents. Lees-Green et al. (2014) investigated the role of calcium-dependent chloride channels, encoded by the ANO1 gene, in initiating the pacemaker potential. These models have been used to explore the dynamics of cellular electrophysiology, however, many models omit this level of detail since the characteristics of the slow wave in aggregate rather than its components are relevant for modeling functional outcomes at the organ level. Many of the computed currents and gating variables in biophysical models can be simplified or lumped depending on the application of the model to reduce the computational load when simulating organ level functional behavior. Therefore, simplified models have often been used to model slow waves in multiscale models. Since ICC contribute the major component of the slow wave, models of ICC are often viewed as analogs of slow wave models, particularly when used as part of multiscale simulations. For reference, simulations of selected ICC models are shown in Figure 1.

Du et al. (2013a) developed a simplified model of gastric ICC by incorporating ionic currents from the Corrias & Buist model into a theoretical model originally developed by Imtiaz et al. (2002). The resulting model achieved a trade-off between biophysical relevance and computational load. This was particularly suited to use in multiscale organ models and has been used to compare *in vivo* recordings with theoretical understandings of slow waves (Wang et al., 2018). Other multiscale organ models use simpler phenomenological models which capture organ-level slow wave dynamics but cannot model cellular ion concentrations at all. For example, an early multi-scale slow wave model used the Aliev et al. (2000) phenomenological cell model to simulate the entrainment of excitable gastric cells (Pullan et al., 2004).

### 2.2. Whole-organ electrophysiology models

Multiscale modeling extends single-cell models by applying governing equations that spatially couple cellular potential. Continuum (multi-domain) modeling and network modeling are the two main approaches for simulating multi-cellular level events. The bidomain model has widely been used to model the propagation of electrical activity across the heart, where investigations have focused on the detailed relationship between tissue structure and function (Austin et al., 2006). Recent advances in microscopic imaging techniques have also allowed the incorporation of 3D imaging into models from the cellular level to the whole-organ level (Sands et al., 2022).

An early example a whole stomach model (Pullan et al., 2004) used an approach where the organ was represented as a continuum-averaged mesh with mesh elements coupled by governing equations. This was the first model of gastric bioelectrical propagation which used the bidomain model. The cell model, in this case the Aliev et al. (2000) cell model, was solved for the continuum element. The bidomain formulation was used as the governing equation, where a reaction-diffusion equation relates the change in cell potential to the flow of current in space and another equation enforces the conservation of charge (Buist and Poh, 2010).

**FIGURE 2:**
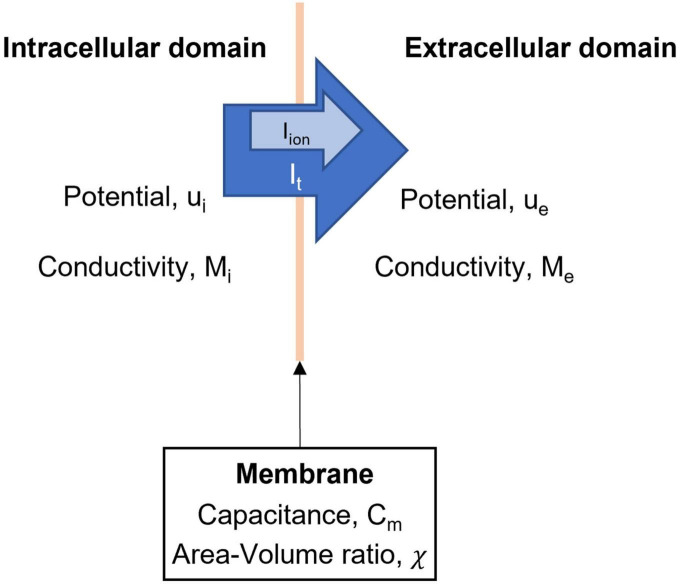
Domains and variables in the bidomain model. The intracellular and extracellular domains are linked by a series of ionic currents, which give rise to changes in membrane potentials of ICC and SMC.

The bidomain model models the flow of current between an intracellular and extracellular domain. The bidomain model was initially extensively used to model the propagation of bioelectrical events across the heart (Geselowitz and Miller, 1983). As noted in Figure 2, the intracellular domain (subscript i) and extracellular domain (subscript e) have conductivity tensors M and potential u. Equations 1 and 3 describe the bidomain model. The physical basis of the bidomain model is that charge in the two domains is conserved. This means that the change in current density must be equal between the intracellular and extracellular domains. Additionally, the changes in current density must travel through the cell membrane, therefore they are equal to the total membrane current (I_t_). Mathematically this is expressed by


(1) $$\nabla \cdot \left(M_{i}\,\nabla u_{i}\right)=\nabla \cdot \left(M_{e}\,\nabla u_{e}\right)=I_{t}$$

where ∇⋅ is the divergence operator and ∇ is the vector gradient operator. Since total membrane current is composed of ionic current (I_ion_) and current due to membrane capacitance, we can form two equations for the bidomain model,


(2) $$\chi \,\left(C_{m}\,\frac{\delta \,u_{t}}{\delta \,t}+I_{i\,o\,n}\right)=\nabla \cdot \left(M_{e}\,\nabla u_{e}\right)$$

(3) $$\chi \,\left(C_{m}\,\frac{\delta \,u_{t}}{\delta \,t}+I_{i\,o\,n}\right)=\nabla \cdot \left(M_{i}\,\nabla u_{i}\right)$$

where χ is the ratio of the surface area to the volume of the integration unit and C_m_ is the capacitance of the membrane.

Two variations on the bidomain model have been used for modeling of gastric electrophysiology. The monodomain model simplifies the bidomain model by assuming that conductivity tensors of the two domains are linearly proportional, this reduces the number of computations required to solve the model at each time step.

Another approach is to model cells as nodes in a network and couple ionic currents as a transfer of charge between nodes or some extracellular space. The coupling is achieved through gap junction models which are typically Ohmic resistor in a network. A recent paper by Ahmed et al. (2021) demonstrated this for gastric cells but the approach has been used in other biological modeling. For example, models of uterine electrophysiology used a grid lattice network with gap junction coupling to explore organ level bioelectrical propagation (Xu et al., 2015). A previous study has also proposed an extended bidomain or “tridomain” model to simulate the sharing of extracellular space by ICC and SMC (Buist and Poh, 2010; Sathar et al., 2015), which would be relevant given the close association between ENS and ICC in the SIP syncytium. A more detailed derivation of both the bidomain model and extended bidomain model for the gastrointestinal context are presented in Buist and Poh (2010).

### 2.3. Electromechanical cell models

**FIGURE 3:**
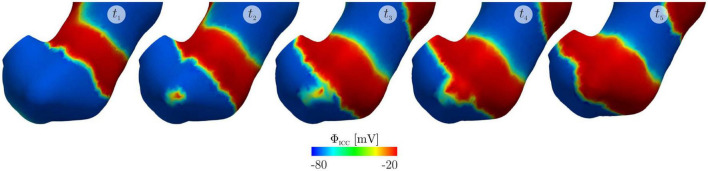
An electromechanical model of gastric slow waves and contraction published by Klemm et al. (2023) reproduced from Klemm et al. (2023) licenced under CC-BY-NC-ND 4.0. The simulations show the emergence of an ectopic pacemaker of slow waves in the gastric antrum due to stretching and the subsequent change in slow waves and mechanical contractions at t1: 60 s, t2: 62 s, t3: 64 s, t4: 65 s and t5: 69 s.

Mechanical contraction has been modeled (Figure 3) by coupling with electrical potential (Du et al., 2011; Brandstaeter et al., 2018; Klemm et al., 2020, 2023) where the relationship between electrical potential and mechanical contraction is modeled as a continuous one. For successful biomechanical modeling the constitutive parameters that define tissue properties need to be determined. Bauer et al. characterized the biomechanical properties of different regions of the porcine stomach wall (Bauer et al., 2020). Their work showed that the stomach wall was spatially heterogeneous and mechanical properties were anisotropic. Reconciliation of this data with anatomical map of muscle thickness and fibre orientation (Avci et al., 2022; Di Natale et al., 2022), will progress work on biomechanical modeling of the stomach through finite element approaches.

Another approach is a meshless smooth particle hydrodynamics approach as demonstrated by Alexiadis et al. (2021) where intestinal contents were modeled. The particles representing intestinal contents interacted with a lattice of distinct particles that represented the intestinal wall. The intestinal wall particles were constrained by linear springs. This model simplified the mechanics of the intestinal wall because the problem was primarily concerned with modeling the velocity of intestinal content flow in normal and diseased intestinal ENS health.

### 2.4. Coupled autonomic-organ models

While no models of stomach motility with vagal neural control have been developed, there are examples in the literature of neural control systems being modeled in combination with motility modeling for other organs of the GI tract. Many of these examples are from the past decade, underlining the novelty of this research domain. Good examples of earlier models of enteric nerve physiology and their impact of motility include the series of models developed by Chambers et al. (Chambers et al., 2008, 2011, 2014a,b), which have also been covered in a separate review article (Chambers et al., 2014b).

The aforementioned model of intestinal contraction and fluid transport by Alexiadis et al. (2021) whose biomechanics approach was described earlier also incorporated the effect of the ENS. Intestinal wall particles were coupled to the output layer of an artificial neural network which enforced the contraction or relaxation of the intestinal wall particle. The computational model implemented a theorized mechanism of peristalsis where the presence of a bolus caused stretching distal to the peristaltic wave and the resulting in a feedback response via the ENS. The ENS was modeled as an artificial neural network, which was trained by reinforcement learning to favor a maximal velocity of intestinal content transport.

Another example is a model of colonic motility by Barth et al. (2017). This model uses multiscale approach, modeling various cell populations separately and connecting them in a network with interactions defined by fitting to experimental measurements. The neural control was implemented as a network of artificial neurons positioned in multiple sub-populations longitudinally along the colon. Sub-populations were positioned such that they resembled the arrangement of neurons observed by imaging of rodent tissue. ICC were simulated using the model proposed by Edwards and Hirst (2006). However, the innervation of ICC by the ENS was not modeled. Instead, only the generation of junction potentials at circular smooth muscle cells was modeled. Neural stimulation in this model resulted in the muscles reaching or failing to reach a contraction threshold when ICC mediated slow wave depolarization occurred.

While there are few GI specific models using coupled neural electrophysiology, smooth muscle interactions with neural stimulation have been modeled for other smooth muscle organs such as the bladder and uterus. The bladder receives central nervous system innervation from the spinal cord. Innervation of the bladder is more direct than the stomach due to the lack of an intrinsic bladder nervous system similar to the ENS. Sympathetic neurons directly synapse with smooth muscle of the bladder to cause excitatory and inhibitory junction potentials (Fowler et al., 2008). The existence of interstitial cells in the bladder is well established, but their role in neurotransmission is an area of ongoing research (Koh et al., 2018).

Computational models of the propagation of electrophysiological activity have been published for both organs. In these examples, published by Appukuttan et al. (2015) and Xu et al. (2015) for the bladder and uterus, respectively, cell-cell coupling by gap junctions was modeled as an Ohmic resistor. Appukuttan et al. (2015) modeled bladder SMCs as passive single compartment cells which responded to external electrical stimulation, including stimulation occurring as a result of synaptic input from neurons. Results showed the time course of the spread of junction potentials induced by synaptic transmission.

In contrast, the model of uterus electrophysiology by Xu et al. (2015) uses a biophysical model of SMCs to demonstrate entrainment of SMC electrophysiological activity in the uterus. This model does not incorporate any neurotransmission, but it does model passive interstitial cells. The authors found that their model was able to simulate entrainment without a defined pacemaker region only if there was sufficient connectivity between adjacent cells.

Finally, Dokos et al. (1996) published a model of neurotransmitter release at the sinoatrial node. The mathematical model published by Dokos et al. (1996) was very detailed, and suitable for theoretical investigation at a cellular scale. The results of their simulations suggested that sustained negative chronotropic effects of cholinergic stimulation were not the result of the time course of neurotransmitter. Instead, the model results suggested that potassium currents controlled the longevity of the chronotropic response. The incorporation of a detailed biophysical model such as this one directly into a whole organ, multi-scale model would be computationally difficult. However, analysis of complex biophysical models can show how they can be simplified to yield new mathematical models suitable for multi-scale computational modeling. The simplified biophysically-based model of ICC gastric slow wave activity by Du et al. (2013a) is an example of this approach.

## 3. Future directions

### 3.1. Models of autonomic control of the stomach

There is clear experimental evidence that autonomic control of gastric motility occurs through the ENS, ICC, and SMC. The key gap in knowledge exists in identifying the details of these how various components, from the network connectivity of cells, to the expression of particular cells, and regional differences within the stomach, interact to bring about a complex and coordinated control of motility under various physiological stimuli. A comprehensive evaluation of these interactions is critical, as every component is a potential target of disease biomarkers or therapies, which may have cascading effects on the tightly co-regulatory system of control. Mirroring the availability of experimental data, a number of biophysically based cell models of ICC and SMC have been developed in recent decades, with further attempts to link them to biomechanical contractions. These investigations provide the critical building blocks for incorporation of neural regulation of GI motility, which has remained relatively crude by assuming direct innervation of SMC without interactions with ICC. The main remaining challenge is a lack of consistency in studies specifically investigating interactions between the ENS, ICC, and SMC and an understanding of the variation in these interactions between functional regions of the stomach and between animal models.

### 3.2. Applications of mathematical models

One potential application of mathematical models is to understand the integrated mechanisms of diseases related to the brain-gut axis. For example, it has been highlighted that in Parkinson’s disease, propagation of pathological α-synuclein aggregates throughout the nervous system, and α-synuclein propagation along the brain-gut axis is a potential disease initiation mechanism (i.e., Braak’s hypothesis) (Braak et al., 2003). There is significant evidence that the vagus and enteric nerves facilitate this propagation in the central nervous system and from the gut, respectively (Fasano et al., 2015). Such changes in the enteric nerves have been proposed as a potential biomarker and treatment target of Parkinson’s disease. However, the details of how neuromodulation influences and normalizes gastric slow wave dysrhythmias remain unclear. An aspect that the modeling can contribute is to develop a detailed structural-functional (neuro-electromechanical) model of stomach, with a vagal neurocircuit model complementing the existing whole-organ gastric slow wave model. The permutations of stimulation protocols and their effects of changes of gastric slow waves and motility can then be tested. This will enable future *in silico* hypothesis testing for optimal neuromodulation parameters targeted at specific symptoms caused by Parkinson’s disease.

Secondary clinical applications can include generating hypothetical gastric signatures for far-field body-surface mapping investigations (Calder et al., 2022). The whole-organ models representing various disease states and responses to neuromodulation parameters can be placed inside a volume conductor to simulate resultant potentials on the body-surface, which can be detected clinically using far-field devices such as body-surface gastric mapping (BSGM) or a superconducting quantum interference device (SQUID) (Kim et al., 2010; Bradshaw et al., 2016; Calder et al., 2022). Given the relatively low signal-to-noise ratio of gastric slow waves compared to cardiac activity, synthetic signals have the advantage of being noise-free so that the true response of the stomach can be explored, and a refined target can be generated for analysis of real signals. Another potential application of the models is to understand the impact of vagotomy on gastric slow waves and motility, which has been shown to significantly alter neuroendocrine peptide levels in the GI tract (El-Salhy et al., 2000), and potentially impacts the response of the stomach to neuromodulation.

One clinical application of computational physiology models is in closed loop control of neurostimulation devices (Payne et al., 2019). Closed-loop control requires the stimulation system to measure a functional quantity and predict the stimulation parameters that will bring the functional quantity towards a desirable set point. The predictive model required for this needs to reach a suitable tradeoff for accuracy and computation speed. The approach taken by Branen et al. (2022) to developing such a model for the cardiovascular system was to use outputs from a mathematical model as training data for several neural networks. The neural networks were used to predict heart rate and mean arterial blood pressure outcomes over hundreds of cardiac cycles. The trained hyperparameters in the neural network model are well suited for the fast prediction of cardiovascular function given inputs for stimulation parameters (pulse width and frequency at three stimulation locations), and the current measured mean arterial pressure and heart rate. The feasibility of this approach for gastric clinical applications is contingent on the availability of a high-quality, predictive, and accurate mathematical model with which to generate valid training data. At present, neuromodulation of the brain-gut axis is receiving active interest, with studies that have reported recovery of gastric functions in functional dyspepsia patients (Zhu et al., 2021) as well as new metrics from non-invasive body-surface gastric mapping studies for definitive of normative values and classifications of diseases (Gharibans et al., 2022; Varghese et al., 2022). Together, these emerging metrics can be used as objective functions for brain-gut axis models to generate targets to device closed-loop protocols for controlling gastric functions via neuromodulation. Closed-loop control of gastric pacing, where electrical stimulation is delivered to the smooth muscle syncytium rather than nerves, has been demonstrated using a model of gastric slow wave propagation (Wang et al., 2022).

## 4. Conclusion

In conclusion, gastric functions are highly dependent on activity from the vagus nerve and enteric nervous system, yet the exact interactions between the gut and brain remain under-investigated. Advances in experimental and computational techniques will lead the development of predictive multi-scale models that can be used to explore the impacts of various pathological conditions related to the brain-gut axis and generate potential treatment targets for the next generation of neuromodulation devices.

## Author contributions

OA, RA, LC, and PD were involved in all aspects of manuscript preparation. All authors contributed to the article and approved the submitted version.
